# Single‐Atom Metallophilic Sites for Liquid NaK Alloy Confinement toward Stable Alkali‐Metal Anodes

**DOI:** 10.1002/advs.202206479

**Published:** 2023-01-16

**Authors:** Junya Cui, Bowen Jin, Annan Xu, Jiale Li, Mingfei Shao

**Affiliations:** ^1^ State Key Laboratory of Chemical Resource Engineering, College of Chemistry Beijing University of Chemical Technology Beijing 100029 China; ^2^ Department of Chemical Engineering, School of Chemistry and Chemical Engineering University of South China Hengyang 421001 China

**Keywords:** chemical confinement, dendrite‐free, liquid anode, NaK alloy, single‐atom

## Abstract

Room temperature liquid NaK alloy is a promising candidate for high performance metal batteries, due to its dendrite‐free property and high energy density. However, its practical application is hindered by the high surface tension of liquid NaK, which causes difficulties in maintaining a stable contact with a current collector. Here, the authors demonstrate the extraordinary stable confinement of NaK alloy at room temperature by constructing a super‐wetting substrate, which is based on highly dispersed cobalt‐single‐atom carbon nanoarrays. The developed liquid anode electrode prevented successfully the leakage of NaK alloy even in harsh stress (>5 MPa) or sharp shock conditions. The symmetric cells achieved ultra‐long reversible plating/stripping cycling life in both Na‐ion (>1010 hrs) and K‐ion electrolytes (>4000 hrs) at 10 mA cm^−2^/10 mAh cm^−2^. Upon fitting with Na_3_V_2_(PO_4_)_3_, the NaK assembled full battery provided high energy density (332.6 kWh kg^−1^) and power density (11.05 kW kg^−1^) with excellent stability after >21600 cycles, which is the best value reported so far. The prepared pouch cell was able to drive a four‐axis aircraft, demonstrating a great prospect in practical application. This work offers a new approach in the preparation of advanced dendrite‐free liquid metal anodes with promising applications in electrochemical energy storage.

## Graphical abstract

The liquid NaK alloy is confined firmly at room temperature on superwetting interface of cobalt‐single‐atom‐based carbon nanoarrays, which prevents the leakage even in harsh stress or sharp shock condition. More remarkably, the as‐assembled full battery shows ultralong cycling lives of up to ≈6000 and 21 500 cycles at 1 and 10 C, respectively.

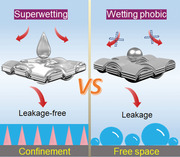

## 1 Introduction

The widespread use of portable electronics and electric vehicles has evoked an enthusiasm to explore reliable and low‐cost energy‐storage devices, which go beyond current commercially available and affordable lithium‐ion batteries with high‐energy density.^[^ ^1^, ^2^ ^]^ Rechargeable metal batteries involving natural abundant elements (e.g., Na and K) are appealing candidates because of their high specific capacities (1166 and 687 mAh·g^−1^ for Na and K, respectively) and low redox potentials (−2.714 and −2.924 V vs standard H_2_ electrode for Na^+^/Na and K^+^/K, respectively).^[^ ^3^, ^4^, ^5^ ^]^ Unfortunately, the uncontrollable dendrite growth of metal anodes during a plating/stripping process causes an internal short of battery, which leads to serious safety issues.^[^ ^6^, ^7^, ^8^ ^]^ Therefore, several strategies have been proposed and applied to suppress the dendrite growth such as interfacial engineering,^[^ ^9^, ^10^ ^]^ electrolyte optimization,^[^ ^11^, ^12^ ^]^ and construction of an artificial solid electrolyte interphase^[^ ^13^, ^14^, ^15^ ^]^; however, the construction of a completely dendrite‐free metal anode for high‐performance batteries is still a challenge.

Liquid metals or alloys have self‐healing functionality that suppresses dendrites generation in the liquid–liquid interface; thus, they are promising materials for overcoming the dendrite growth issue.^[^ ^16^, ^17^, ^18^ ^]^ NaK alloy with the merits of low molten point, high electronic conductivity, high energy density, and low reduction potential is a promising candidate for the liquid anode.^[^ ^19^, ^20^ ^]^ However, the construction of a practical stable liquid electrode with a NaK alloy that is contacted well with a current collector is challenging, due to its high fluidity nature.^[^ ^21^, ^22^ ^]^ Moreover, the high surface tension of a liquid NaK alloy delivers a poor wetting property, which makes it difficult to realize fluidic NaK alloy confinement in a conducting substrate.^[^ ^23^ ^]^ Therefore, considerable efforts have been conducted by means of “external force” such as high temperature and high pressure. For instance, Goodenough and co‐workers showed a decrease in the surface tension of liquid NaK alloys under a high temperature of 420 °C, which facilitated the immobilization of NaK on the substrate.^[^ ^24^ ^]^ In another study from the same group, a vacuum infiltration technique for squashing NaK alloy into an aluminum and copper foam at room temperature was developed.^[^ ^25^ ^]^ Nevertheless, the as‐fixed liquid NaK alloy was leaked easily in the harsh conditions of stress or vibration when external physical conditions were removed. Thus, it is desirable to explore mild and universal solutions that will enable firm confinement of a liquid metal into substrates at room temperature.

**Scheme 1:**
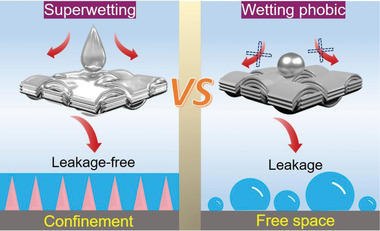
Schematic illustration of liquid NaK Alloy confinement.

In this work, we develop a new chemical strategy to confine firmly a NaK alloy at room temperature by constructing a super‐wetting interface of cobalt–single‐atom‐based carbon nanoarrays (Co‐SACN) on the current collector (**Scheme** **1**). Benefiting from the strong metallophilic and adequate confined space, the Co‐SACN successfully prevented the leakage of the NaK alloy even in harsh stress (e.g., >5 MPa) or sharp shock condition. The nanoarrays can be customized into various substrates from one‐dimensional wires to three‐dimensional foams. Wettability test and theoretical calculation confirmed that the K atom in the NaK alloy adsorbed preferentially on the Co‐SACN. K also formed strong chemical bonds with Co‐SACN. The resulting Co‐SACN@NaK anode exhibited a highly stable plating/stripping process with long service times of >1010 and 4000 h in NaClO_4_ and KPF_6_ electrolytes, respectively at 10 mA cm^−2^/10 mAh cm^−2^. Remarkably, the assembled NaK||Na_3_V_2_(PO_4_)_3_ battery showed ultralong cycling lives of up to ≈6000 and 21500 cycles at 1 and 10 C, respectively. The battery was working for more than a year, and still working. In addition, a negligible capacity attenuation was observed even at 30 C. The battery also showed better stability compared with previous reports. Moreover, the proposed pouch cell was able to power a four‐axis aircraft without series (3.2 V), showing the promising practical application of the strategy.

### 1.1 Fabrication and Characterization of Co‐SACN@NaK

**Figure 1:**
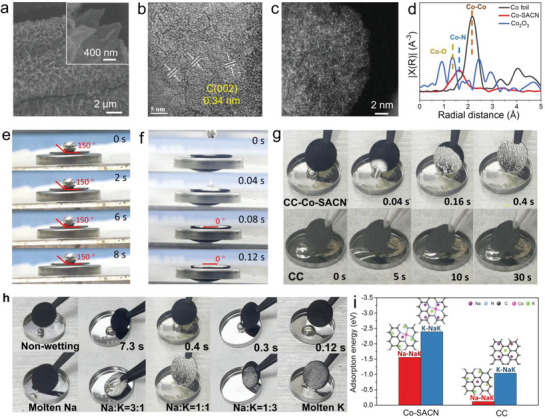
Characterization of Co‐SACN and Co‐SACN@NaK. a) SEM (inset: high magnification image), b) HRTEM and c) AC‐HAADF‐STEM images of Co‐SACN. d) FT‐EXAFS spectra of Co‐SACN, Co foil, and Co_2_O_3_. Dynamic contact angles of NaK alloy on: e) CC and f) Co‐SACN. g) Wetting process of NaK alloy on CC and CC/Co‐SACN. h) Wetting process of NaK (different proportion) alloy on Co‐SACN. i) The adsorption energy of NaK alloy on CC and Co‐SACN; Na atom and K atom are used as adsorption atoms, respectively.

The Co‐SACN was synthesized by topological transformation of Co‐zeolitic‐imidazolate‐frameworks (ZIF‐67) nanosheets arrays under a thermal annealing process in nitrogen (Figure S1, Supporting Information). The prepared carbon cloth (CC)/Co‐SACN showed a hierarchical nanosheet arrays morphology in its scanning electron microscopy images (**Figure** **1**a). Its high‐resolution transmission electron microscopy (HRTEM) images suggest obvious lattice fringes with a spacing of 0.334 nm, which is ascribed to the (002) plane of graphitized carbon (Figure 1b and Figure S2, Supporting Information).^[^ ^26^ ^]^ The X‐ray diffraction (XRD) pattern exhibits the absence of cobalt characteristic reflections, suggesting an atomic‐dispersed nature (Figure S3, Supporting Information). Uniform distribution of Co elements with C and N elements is confirmed by energy‐dispersive X‐ray (EDX) elemental mappings (Figure S4, Supporting Information). The spherical aberration‐corrected high‐angle annular dark‐field scanning TEM (AC‐HAADF‐STEM) image shows the uniform distribution of high‐density isolated single Co atoms (white dots) on the substrates (Figure 1c).

The high‐resolution Co 2*p* spectrum of the prepared Co‐SACN shows strong peaks at 780.9 and 796.5 eV, which are attributed to the oxidation state rather than metallic state of Co species, demonstrating its isolated coordination configuration (Figures S5 and S6, Supporting Information). And the concentration of Co atom in Co‐SACN is 0.34% (Table S1, Supporting Information). The N 1*s* spectrum exhibits the presence of pyridinic N, pyrrolic N, and graphitic N species at 398.2, 400.8, and 403.1 eV, respectively. The electronic structures and coordination environment of Co atoms are further investigated by K‐edge X‐ray absorption near‐edge structure spectroscopy (XANES) and extended X‐ray absorption fine structure (EXAFS) (Figure 1d). The white line of CC/Co‐SACN shifts toward a higher position relative to the Co foil, indicating a positive‐charge state of Co atoms in the Co‐SACN (Figure S7, Supporting Information). This is in agreement with the XPS result. Moreover, the Fourier‐transformed k3‐weighted EXAFS profile displays a strong signal at ≈1.61 Å, corresponding to the Co‐N/C coordination on the first shell. Meanwhile, Co–Co coordination and Co–O coordination at 2.17 and 1.36 Å, respectively, are not observed. These results confirmed the atomic dispersion of isolated Co atoms in the prepared Co‐SACN, which is consistent with the AC‐HAADF‐STEM results.

It has been shown that liquid NaK alloy was hardly confined into a traditional substrate without “external force” due to its high surface tension.^[^ ^23^ ^]^ This is confirmed by the high contact angle of ≈150° for the CC substrate and CC/ZIF‐67 found in this study (Figure 1e and Figure S8, Supporting Information). Moreover, the bare CC likely excluded the NaK alloy in the contact angle measurement, indicating the high metallophobic behavior of the CC substrate (Figure 1g and Video S1, Supporting Information). Intriguingly, the prepared CC/Co‐SACN shows a superwettability behavior with the NaK alloy and exhibited a significantly small contact angle of <2° in 0.04 s (Figure 1f and Video S2, Supporting Information). The liquid metal is “sucked” into the CC/Co‐SACN within 0.4 s upon contact with the NaK alloy at room temperature (Figure 1g). To investigate the mechanism of the wetting process and confinement effect, infiltration measurements are performed (Figure 1h and Videos [Link], [Link], [Link], [Link], [Link], Supporting Information; NaK: 10 mg). It can be observed that the infiltration rate of the liquid metal is associated with the ratio of Na and K. The pristine molten Na at 100 and 300°C was difficult to absorb into the CC/Co‐SACN (Video S8, Supporting Information). However, the infiltration time decreases rapidly from 7.3 to 0.3 s with the increase in the K content from 25 to 75 wt% in the NaK alloys. For pure molten K at 100 °C, the liquid invades quickly into the CC/Co‐SACN substrate just in 0.12 s, indicating the strong interaction between K atom and Co‐SACN. Moreover, the superior wetting ability of Co‐SACN can also be extent to other single‐atom‐based carbon nanoarrays or materials contain with single‐atom‐based carbon nanoarrays, e.g., Zn‐SACN, Co‐NC (Figure S9, Supporting Information). However, as for the bare CC or N‐riched carbon substrate without single atoms, the molten K exhibits wetting phobic properties, indicating the important of Co‐SACN centers on the wetting process (Figure S10, Supporting Information). Density functional theory is then carried out to understand the interface interaction between Co‐SACN and NaK alloy. The absorption energy (*E*_ads_) of NaK (Na:K = 1:1) on CC surface and Co‐SACN is shown in Figure 1i. K and Na atoms are used as adsorption units, respectively. Remarkably, lower *E*_ads_ is observed in the Co‐SACN surfaces than the bare CC of both Na and K atom in the NaK alloy. In particular, the K is adsorbed easily on the Co‐SACN with the lowest *E*_ads_ of −2.39 eV, which is consistent with the infiltration experiments. These results suggest clearly the strong interaction between the Co‐SACN interface and K atom of the NaK alloy, which leads to the fast infiltration process.

### 1.2 Confinement Ability and Electrochemical Performance of Co‐SACN@NaK

**Figure 2:**
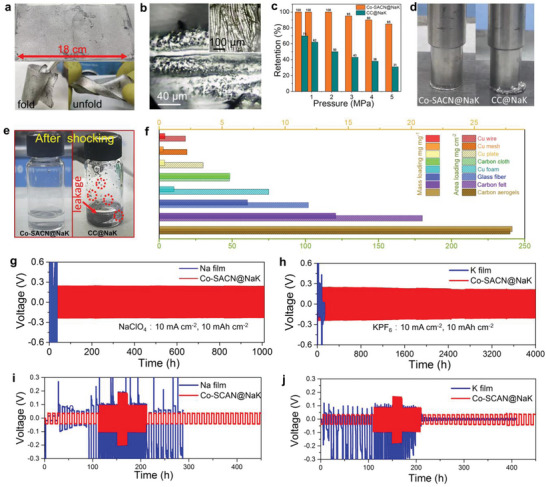
Characterization and electrochemical performance of Co‐SACN@NaK. a) Optical and b) confocal microscope images of Co‐SACN@NaK. c) Stress tests of Co‐SACN@NaK and d) optical image of Co‐SACN@NaK and CC@NaK under 5 MPa. e) Optical image after shocking tests in PC. f) NaK mass loading of different electrodes. Long cycling performance of Co‐SACN@NaK‐SC at 10 and 10 mA cm^–2^ in: g) Na ion electrolyte and h) K ion electrolyte. The rate performance of Co‐SACN@NaK, Na‐, and K‐foil‐based: i) Na and j) K symmetrical cells at 10 mAh cm^–2^ with a current density of 2–10 mA cm^–2^.

Benefiting from the superwetting property of the Co‐SACN@NaK, a large area (10 cm × 18 cm) of the material with high flexibility can be prepared easily (**Figure** **2**a), which is also promising for scale‐up production of the material. The surface and inner space of the Co‐SACN nanoarrays framework was covered fully with NaK alloy (Figure 2b). Moreover, almost leak‐free appearance is observed for the Co‐SACN@NaK even at a high pressure of 5 MPa by the squeeze test (Figure 2c,d), which indicates extreme stability. Similarly, liquid metal beads are not formed in the electrolyte after 100 h of violent oscillation in a propylene carbonate (PC) electrolyte (Figure 2e) or diethyl carbonate (DEC)/ethylene carbonate (EC) electrolyte (Figure S11, Supporting Information). The absence of the beads reduces the risk of short circuit during battery assembly. In contrast, the liquid NaK is easily detached from the pristine CC substrate under pressure and shock condition, albeit it was distributed to the bare CC network via vacuum infiltration. Benefiting from the simple growth of Co‐SACN on various substrates, conductive substrates (e.g., Cu with different shapes of wire, mesh, plate, and foam), carbon substrates (e.g., felt and aerogels), and even insulating substrates (e.g., glass fiber) were used in hosting NaK alloys (Figure S12, Supporting Information). Optical microscopy photographs exhibit that the NaK alloy covers uniformly the skeletons of the above substrates, enabling the construction of various types of batteries (e.g., fiber battery, flexible battery, coiled battery, and solid‐state battery). Moreover, the mass loading of NaK alloy can be increased from 18 to 240 mg cm^−2^ (0.45–28 mg mg^−1^) using multidimensional electrodes (Figure 2f). This guaranteed the high energy output of the developed batteries.

The electrochemical properties of the prepared Co‐SACN@NaK electrode is determined using the symmetric cells (SC) tests in Na‐ion electrolyte (1 m NaClO_4_:PC with 5% fluoroethylene carbonate (FEC)) and K‐ion electrolyte (1 m KPF_6_:EC/DMC with 5% FEC). The SACN@NaK‐based symmetric cell provides a long‐term plating/stripping process (1010 h) with smooth voltage plateaus of ≈250 mV in the NaClO_4_ electrolyte at 10 mA cm^−2^ and 10 mAh cm^−2^ (Figure 2g). In contrast, the Na‐foil‐based symmetric cell exhibits a high overpotential of ≈400 mV at the initial cycles, but its sudden raises over 4.9 V and the cell explodes after 36 h due to dendrite accumulation and pulverization of Na (Figure S13, Supporting Information). More impressively, the Co‐SACN@NaK‐SC exhibits a promising reversible stripping/plating ability in the K‐ion‐based electrolyte (Figure 2h), which delivers a stable voltage plateau at ≈230 mV) even after ultrahigh cycling for >4000 h at 10 mA cm^−2^ and 10 mAh cm^−2^. In contrast, the K‐foil‐based symmetric K||K cell provides large voltage fluctuations and short working hours of <130 h. Moreover, the Co‐SACN@NaK shows a good rate performance in both the Na‐ion and K‐ion electrolyte. As shown in Figure 2i, Co‐SACN@NaK delivers low voltage hysteresis at 2–10 mA cm^−2^ in the range of 33–190 and 32–180 mV in the Na‐ion and K‐ion electrolytes, respectively (Figure 2i), indicating high reversibility (Figure 2j). The optical microscopy photograph confirms dendrite‐free NaK alloy is still attached tightly with after cycling (Figure S14, Supporting Information). In contrast, both Na‐ and K‐foil‐based cells deliver violent voltage fluctuations and short circuited expeditiously due to dendrite growth. The high performance was very comparable with the best reported Na anode under high current densities and areal capacities (Table S2, Supporting Information).

### 1.3 Full Battery Performance

**Figure 3:**
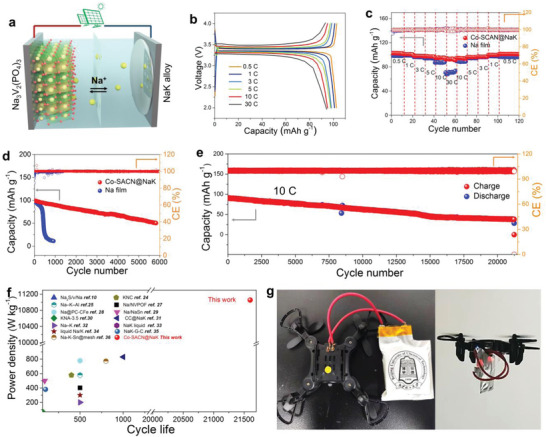
Electrochemical performance of Co‐SACN@NaK full battery. a) The scheme of assembled full battery. b) Voltage profiles and c) rate‐performance of NaK‖Na_3_V_2_(PO_4_)_3_ and Na‖Na_3_V_2_(PO_4_)_3_ at different rate. d,e) Long‐term cycling performance of NaK‖Na_3_V_2_(PO_4_)_3_ at 1 and 10 C. f) Comparison of the performance between this work and references.^[^ ^10^, ^24^, ^25^, ^27^, ^28^, ^29^, ^30^, ^31^, ^32^, ^33^, ^34^, ^35^, ^36^ ^]^ g) Digital images of pouch format battery driving four‐axis aircraft.

To meet practical applications, a liquid metal full battery is assembled using Na_3_V_2_(PO_4_)_3_ and NaK as cathode and anode, respectively (**Figure** **3**a). Both NaK||Na_3_V_2_(PO_4_)_3_ and Na||Na_3_V_2_(PO_4_)_3_ batteries show similar voltage profiles with a narrow voltage gap and ultraflat plateau of 3.35 V at a charging rate of 0.5 C (1 C = 110 mAh g^−1^) (Figure 3b and Figure S15, Supporting Information). Intriguingly, the voltage profiles are almost overlapped in NaK||Na_3_V_2_(PO_4_)_3_ when the charging rate is increased from 0.5 to 30 C. The corresponding rate performance also shows that NaK||Na_3_V_2_(PO_4_)_3_ delivers a better rate performance than Na||Na_3_V_2_(PO_4_)_3_ (Figure 3c). The capacity retention of >85% (91 and 106 mAh g^−1^ at 0.5 C) is maintained in NaK||Na_3_V_2_(PO_4_)_3_ at 30 C (charging within 2 minutes), indicating excellent mass and charge‐transfer dynamics in Co‐SACN@NaK. In contrast, the Na||Na_3_V_2_(PO_4_)_3_ cell provides a low‐capacity retention of 60% at 30 C. The assembled NaK||Na_3_V_2_(PO_4_)_3_ battery shows a competitive maximum energy density and maximum power density of 332.6 Wh kg^−1^ (power density = 0.36 kW kg^−1^) and 11.05 kW kg^−1^ (energy density = 286 Wh kg^−1^), respectively. Moreover, NaK||Na_3_V_2_(PO_4_)_3_ shows an ultralong cycling life of >5900 cycles at 1 C with a high CE of >99.9% (Figure 3d). However, the bare Na||Na_3_V_2_(PO_4_)_3_ delivers much inferior electrochemical cycling properties, but dropped suddenly to ≈15 mAh g^−1^ after 400 cycles due to the repeated growth/corrosion of Na dendrites. Notably, the NaK||Na_3_V_2_(PO_4_)_3_ shows an ultralong cycling life of >21 600 cycles with a low‐capacity decay of 0.0027% per cycle and high CE of ≈99.6% at a high charging rate of 10 C (Figure 3e), which is the best among all Na metal batteries. Both batteries operated at 1 and 10 C are still working in our laboratory. In addition, the battery also delivers an initial capacity of 100 mAh g^−1^ with a high mass loading of ≈8.8 mg cm^−2^ at 1 C, which has catch up commercial batteries (Figure S16, Supporting Information). The excellent stability and mass transfer kinetics of Co‐SACN@NaK improved the cycle life and power density of the fabricated battery, which was considered superior to other previously reported Na and liquid NaK metal batteries (Figure 3f).

In addition, the Co‐SACN@NaK electrode can be easily applied as potassium metal anode. Prussian blue analog (PBA) as potassium ion battery cathode was synthesized and its characterization are shown in Figure S17 (Supporting Information). Stable symmetric voltage profile with small hysteresis is observed in Co‐SACN@NaK||PBA cell in a KPF_6_ electrolyte (Figure S18, Supporting Information). A high reversible capacity of 80 mAh g^−1^ with 70% retention is observed even after 2000 cycles, which is much superior than K||PBA battery. To further explore the potential application, an individual NaK||Na_3_V_2_(PO_4_)_3_ pouch cell with a large area (2 cm × 5 cm) was fabricated and used continuously to power a light‐emitting diode wristband with a high open‐circuit voltage of 3.2 V (Figure S19, Supporting Information). Even after being folded repeatedly, the pouch cell showed a high capacity due to the high fluidity of NaK alloys (Figure S20, Supporting Information). Subsequently, a prototype of NaK||Na_3_V_2_(PO_4_)_3_ cells in parallel was fabricated, which is able to drive a four‐axis aircraft (Figure 3g and Video S9, Supporting Information). The above results demonstrate the promising practical application for Co‐SACN@NaK anode in advanced energy‐storage devices.

## 2 Conclusion

We demonstrated the facile and effective construction of highly stable NaK alloy liquid anode at room temperature by a chemical confinement strategy. The atomic‐dispersed Co‐SACN served as an electron‐rich donor that enabled easier adsorption of K atom in NaK alloy. Thus, it was found to be a critical component that attributed to the fast infiltration process. Due to the confinement effect of Co‐SACN, a completely leak‐free NaK liquid is observed even at a high pressure of 5 MPa. Ultralong service times are observed in both NaClO_4_ and KPF_6_ electrolytes for the dendrite‐free Co‐SACN@NaK symmetric cell. When paired with Na_3_V_2_(PO_4_)_3_, the assembled liquid NaK alloy battery achieves high‐energy density with extremely long cycling life of >21 600 (more than a year). These findings provide new insights into the design of advanced alkali liquid metal anodes for next‐generation energy‐storage systems.

## 3 Experimental Section

### Preparation of Co‐SACN

Before growth of Co‐SACN, various substrates were pretreated as followed: carbon‐based substrate (e.g., carbon cloth, carbon felt) was immersed in 0.5 m KMnO_4_ for 20 min and washed by deionized water before used; metal substrate (e.g., Cu wire, Cu foil, Cu mesh, and Cu foam) was etched with 2 m HCl to remove the oxide layer and immersed in propanone, absolute ethanol, and water, respectively; melamine sponge and glass fiber substrate could be used directly. Co‐SACN precursor was fabricated prepared by an aqueous precipitation method. Then, 50 mL of Co(NO_3_)_2•_6H_2_O (0.73 g) solution was quickly added to 50 mL of dimethyl imidazole (1.643 g) solution under magnetic stirring. The above transparent solution converted to purple suspension, after 2 min reaction. Following, the pretreated substrate was immersed into suspension solution for 2 h at room temperature to format Co‐zeolitic‐imidazolate‐frameworks (ZIF‐67, precursor), which were then pyrolysis under N_2_ at 700 °C. After cooled to room temperature naturally, the sample was etched by acid etching (3 m HCl) for 6 h to remove Co metal, and then thoroughly washed using deionized water.

### Preparation of Zn‐SACN

The synthesis process of Zn‐SACN was similar as Zn‐SACN, where Zn(NO_3_)_2•_6H_2_O (0.72 g) solution replaced Co(NO_3_)_2•_6H_2_O and mixed with 50 mL of dimethyl imidazole (1.643 g) solution to formation of ZIF‐8 precursor. Then, the pyrolysis and etching processes for making Zn‐SACN were same as Co‐SACN.

### Preparation of NaK Alloy

Liquid NaK alloy was prepared by physically mixing at room temperature in argon‐filled glovebox (Notice: NaK liquid alloy was explosive in air!). Typically, Na metal and K metal were mixed with different proportions (Na:K = 3:1, Na:K = 1:1, Na:K = 1:3; weight ratio) and transferred to glass vial. Unless otherwise specified, the ratio of NaK for tests was 1:1. The whole process was conducted in an argon‐filled glovebox.

### Preparation of Co‐SACN@NaK Alloy

The Co‐SACN@NaK was prepared in superwetting process. Typical Co‐SACN was contacted with NaK alloy in an argon‐filled glovebox. The metal mass loading in CC/Co‐SACN@NaK for compression and shocking tests were ≈20 mg cm^−2^. The mass loading for electrochemical tests were ≈20 mg cm^−2^. In particular, the mass loading for full cell tests were ≈10 mg cm^−2^. The CC@NaK electrode was synthesized by immersing CC in NaK alloy and followed by a full vacuum process with a mass loading of ≈15 mg cm^−2^.

### Preparation of Na_3_V_2_(PO_4_)_3_ Cathode

To prepare the Na^+^ cathode, Na_3_V_2_(PO_4_)_3_ powder (Shenzhen kejing Cot.), super P, and PVDF were first added to *N*‐methyl pyrrolidone (8:1:1 weight ratio) and milled for 6 h to form a homogeneous slurry. The resultant slurry was cast onto Al foil or stainless‐steel mesh and dried under vacuum condition at 120 °C for 24 h.

### Preparation of PBA‐Positive Electrode

PBA was synthesized according to the previous report.^[^ ^37^ ^]^ First, 40 mL of FeCl_3_ solution (2 mmol) was added to 160 mL of K_4_Fe(CN)_6_ solution (1 mmol) under stirring conditions, which immediately produced precipitation. After 24 h of reaction, dark blue precipitates were collected by centrifugation, followed by vacuum drying at 80 °C to obtain PBA. Then, PBA, super P, and PVDF were added to NMP (8:1:1; weight ratio) for preparing slurry. The resultant slurry was cast onto Al foil or stainless‐steel mesh and dried under vacuum condition at 120 °C for 24 h.

### Characterization

The morphology images were recorded on Zeiss SUPRA55 scanning electron microscope (SEM), which combined with energy‐dispersive X‐ray spectroscopy (EDX), and transmission electron microscopy (TEM) (Philips Tecnai 20 and JEOL JEM‐2010 high‐resolution TEM). The optical image was performed on a Leica DMI8 fluorescence microscope. X‐ray diffraction (XRD) data were performed on Shimadzu XRD‐6000 X‐ray diffractometer (Cu K*α* radiation (0.154 nm) at 40 kV, 30 mA, and scanning rate of 10° min^−1^). X‐ray photoelectron spectra (XPS) were performed on a Thermo VG ESCALAB 250 X‐ray photoelectron spectrometer (pressure: 2 × 10^–9^ Pa; excitation source: Al K*α* X‐rays). The aberration‐corrected high‐angle annular dark‐field scanning transmission electron microscopy (HAADF‐STEM) was performed on FEI Titan Cubed Themis G3 300. The Co K‐edge XAS data were collected at the beamline 1W1B of the Beijing Synchrotron Radiation Facility (BSRF), Institute of High Energy Physics (IHEP), and Chinese Academy of Sciences (CAS). Contact angle was performed by the sessile drop method in glove box. Crimping and shocking tests were carried out by tablet press (MTI, China) and vortex mixer (TAT, China).

### Electrochemical Measurements

The CR2032 coin‐type cells were assembled with Co‐SACN@NaK electrodes and glass paper (or Na/K foil) for symmetrical cell tests with 60 µL electrolyte (Na, 1 mol L^‒1^ NaClO_4_ in PC with 5% FEC; K, 1 mol L^‒1^ KPF_6_ in EC/DEC = 1:1). For full‐cell tests, mass loading of Na_3_V_2_(PO_4_)_3_ and PBA in cathode were 1.0 to 8.8 mg cm^–2^. The galvanostatic charge–discharge curves were conducted on CT2001A cell test instrument (LAND Electronic Co. Ltd) with the voltage window of 2–4 V (vs Na^+^/Na, K^+^/K) under room temperature.

### Preparation of Pouch Na_3_V_2_(PO_4_)_3_ Battery

First, the Na_3_V_2_(PO_4_)_3_ slurry was coated on stainless steel mesh (mass loading: 1.0 mg cm^−2^) as cathode (2 cm × 5 cm for flexible pouch cell; 2 cm × 3 cm for high‐energy density battery). Aluminum and nickel strips were attached as electrode tabs to the sides of the cathode and anode, respectively. A total of 4 mL of electrolyte was injected into the bag followed by a vacuum packaging with a vacuum‐sealing machine (Joyoung Co. Ltd). Particularly, four pieces of cathode and anodes were assembled in aluminum plastic film pouch for high‐energy density battery.

## Conflict of Interest

The authors declare no conflict of interest.

## Supporting information

Supporting Information

Supplemental Video 1

Supplemental Video 2

Supplemental Video 3

Supplemental Video 4

Supplemental Video 5

Supplemental Video 6

Supplemental Video 7

Supplemental Video 8

Supplemental Video 9

## Data Availability

The data that support the findings of this study are available from the corresponding author upon reasonable request.
