# Supplementary material for: Single‐Atom Metallophilic Sites for Liquid NaK Alloy Confinement toward Stable Alkali‐Metal Anodes
Source: Adv Sci (Weinh). 2023 Jan 16;10(8):2206479. doi: 10.1002/advs.202206479 (PMC10015853; doi:10.1002/advs.202206479)
Supplement: Supplementary file 1 — Supporting Information [file ADVS-10-2206479-s010.pdf]

ADVANCED SCIENCE
Open Access

Supporting Information

for *Adv. Sci.*, DOI 10.1002/advs.202206479

Single-Atom Metallophilic Sites for Liquid NaK Alloy Confinement toward Stable Alkali-Metal Anodes

*Junya Cui, Bowen Jin, Annan Xu, Jiale Li and Mingfei Shao**

Supporting Information

Single-Atom Metallophilic Sites for Liquid NaK Alloy Confinement towards Stable Alkali-Metal Anodes

Junya Cui[1,2], *Bowen Jin*[1], *Annan Xu*[1], *Jiale Li*[1] *and Mingfei Shao*[1,]*

[1] State Key Laboratory of Chemical Resource Engineering, Beijing University of Chemical Technology, Beijing 100029, China

[2] School of Chemistry and Chemical Engineering, University of South China, Hengyang 421001, China

*Corresponding authors. E-mail addresses: shaomf@mail.buct.edu.cn (Mingfei Shao)

1.Method

Energy density and power calculation: The energy density and power density of pouch cell is calculated by the following equations:

$$E = \frac{\int UI\mathrm{d}t}{m}$$

$$W = \frac{\int UI\mathrm{d}t}{mT}$$

where *E* is the energy density; *W* is the power density; *I* is the discharging current; d*t* is the time differential; *T* is the discharge time; *m* refers to the total mass of electrodes, electrolyte and separator.

Computational Method: DFT based on CASTEP in the materials Studio 8.0 software package (Accelrys Software Inc., San Diego, CA). DFT plane wave pseudopotential is also used. The electronic exchange and correlation potential are described by the Perdew–Burke–Ernzerhof (PBE) function of the generalized gradient approximation (GGA). An energy cutoff of 340 eV, 3×3×1 k-point sampling in the surface Brillouin zone is used in the calculations. When optimizing the

model, the following convergence criteria are used: (1) 1×10^{-5} ev/atom convergence energy; (2) the converge criteria of the force on each relaxed atom below 0.05 eV/Å were used; (3) Movement convergence of 1×10^{-3} Å. The DFT+U method evaluated the on-site coulomb interactions in the localized d orbital and exchange interactions by adding an effective Hubbard-U parameter to express the repulsion between electrons on the same orbital. In this work, the value of Hubbard-U parameter was set to 3.5 eV for Co atom.

2. Supplementary Figures and Table

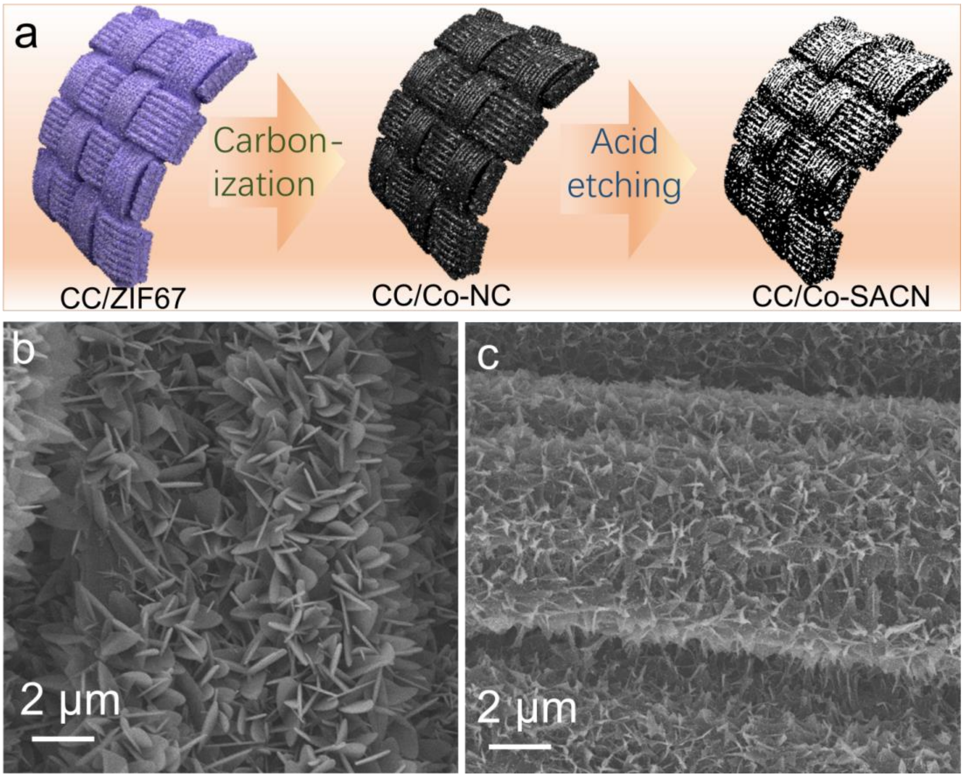

Figure S1. a) Schematic diagram of preparing process of Co-SACN. SEM images of (b) CC/Co-NC and (c) Co-SACN.

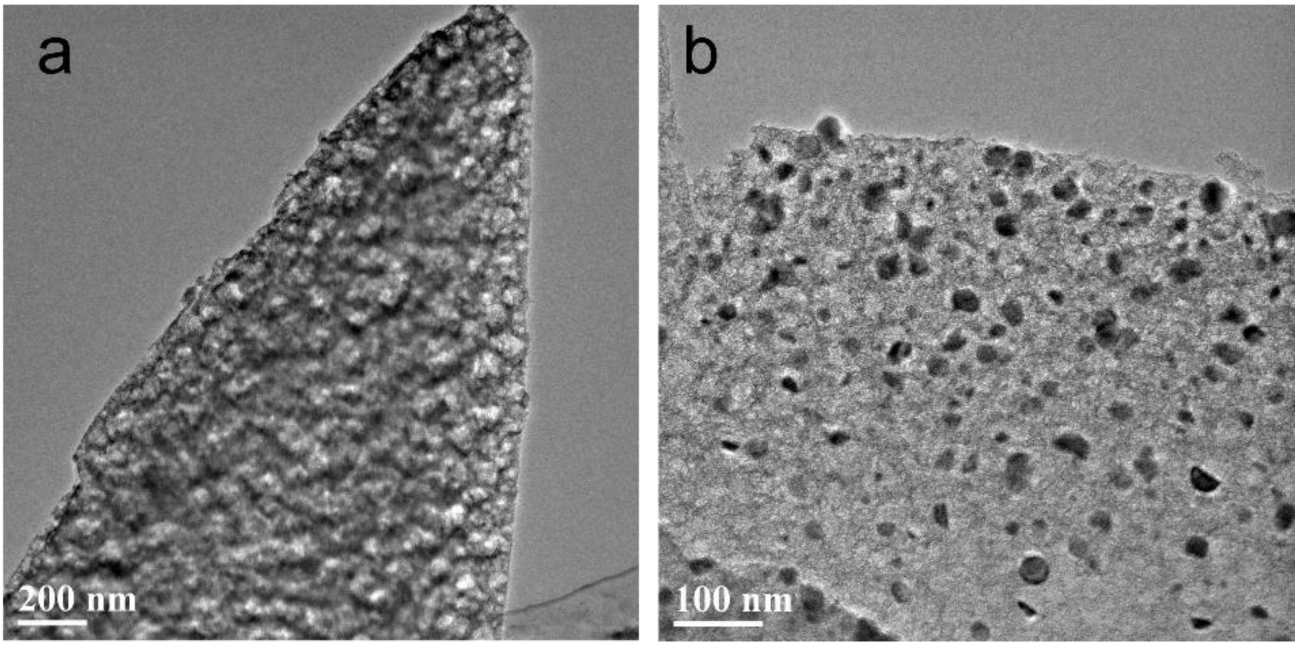

Figure S2. TEM images of (a) Co-SACN and (b) Co-NC.

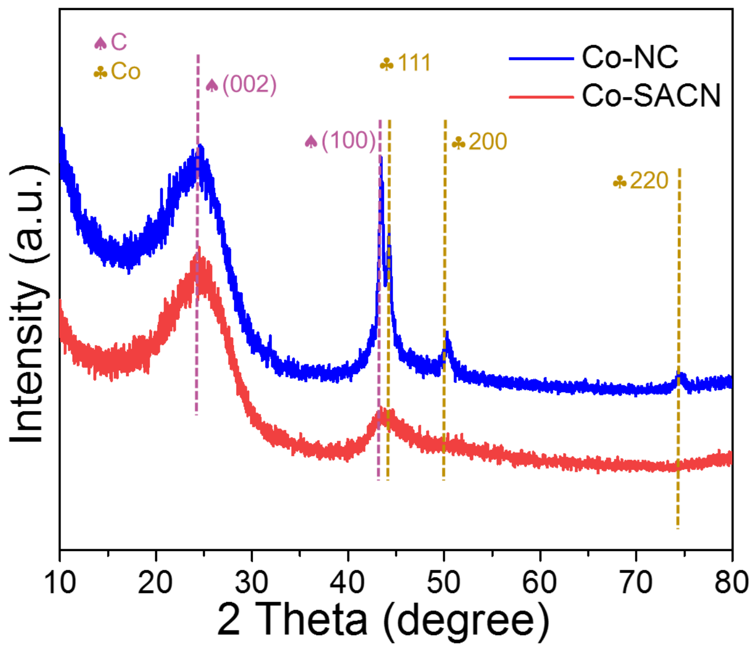

Figure S3. XRD pattern of Co-NC and Co-SACN.

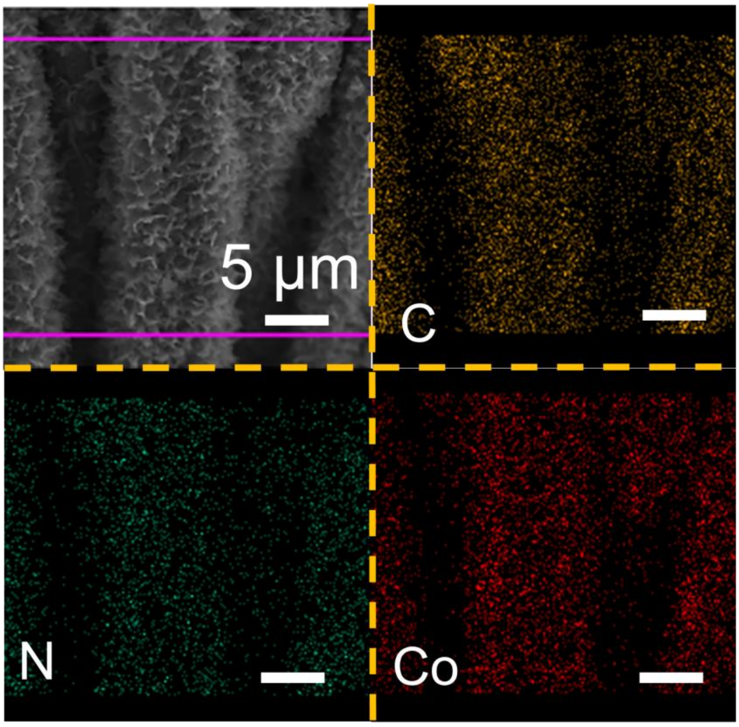

Figure S4. SEM image and corresponding EDX-mapping images of Co-SACN.

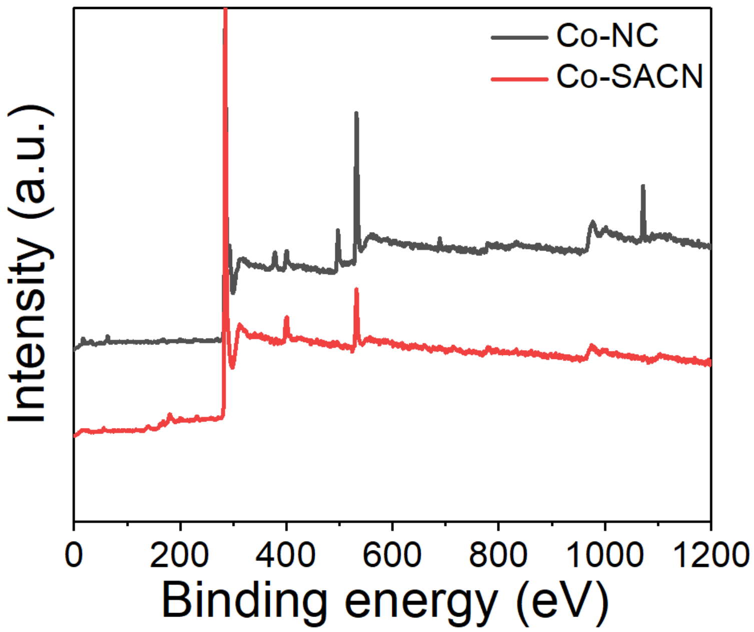

Figure S5. XPS spectra of Co-NC and Co-SACN.

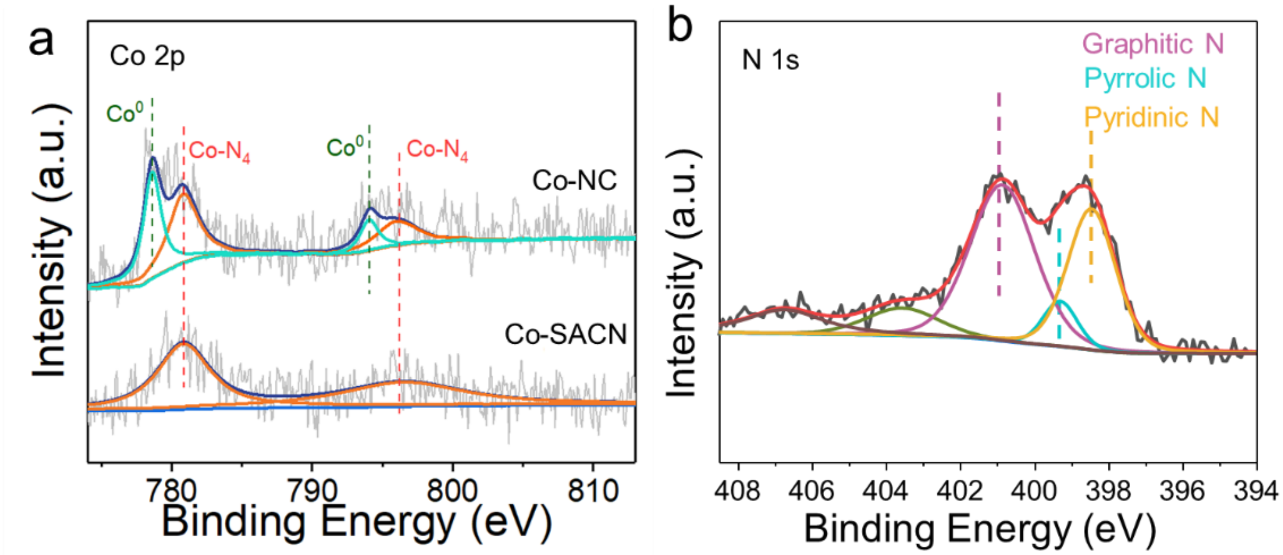

Figure S6. High resolution XPS (a) Co 2*p* and (b) N 1*s* spectrum of Co-SACN.

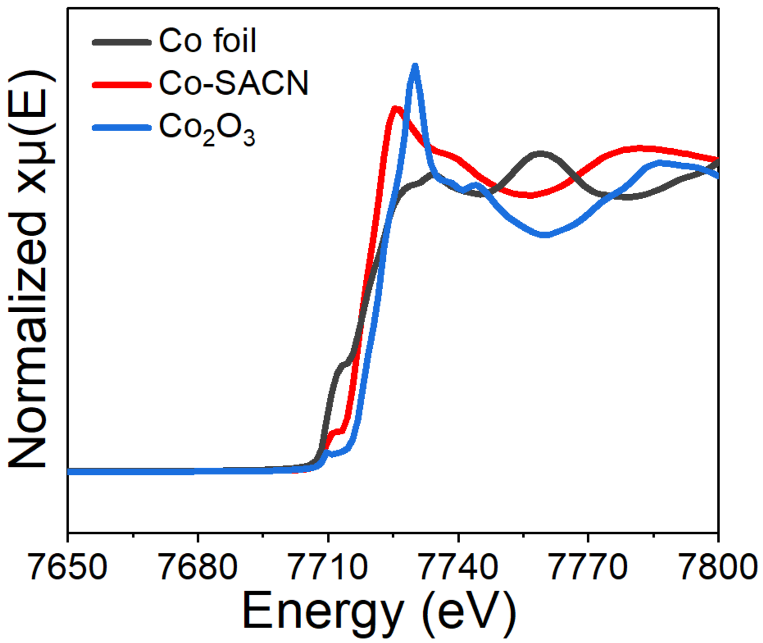

Figure S7. XANES spectra of Co foil, Co-SACN and Co_2O_3.

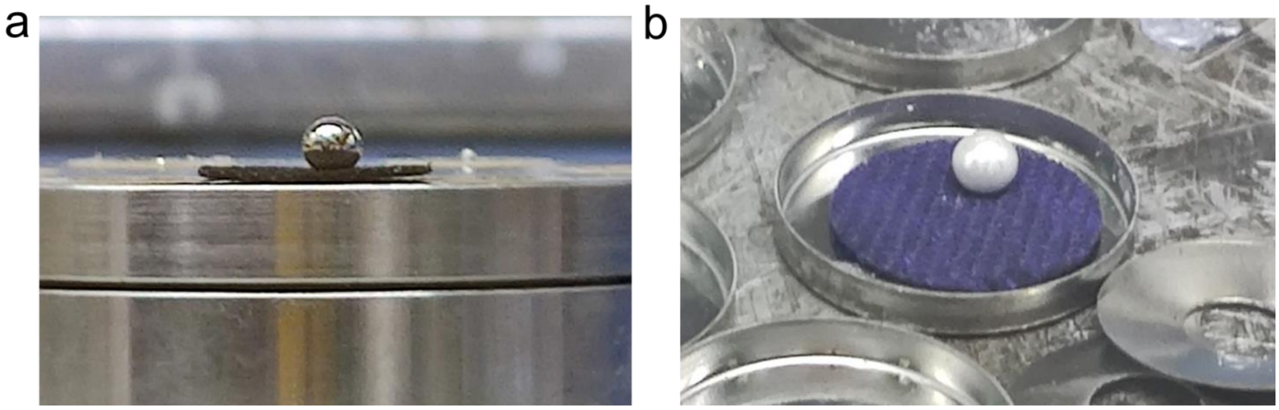

Figure S8. Contact angle measurement of NaK on (a) CC and (b) CC-ZIF67.

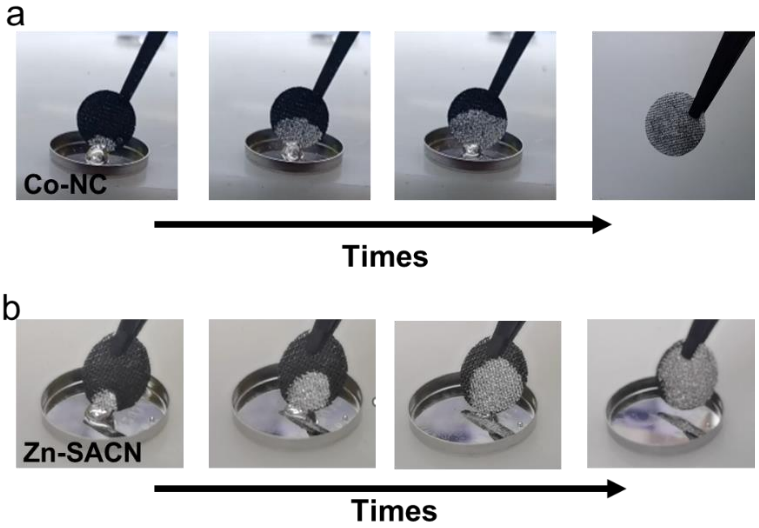

Figure S9. Wetting process of NaK alloy on a) Co-NC b) Zn-SACN.

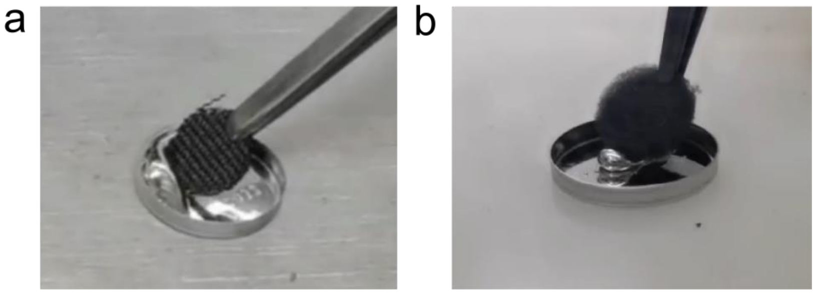

Figure S10. Contact molten K with a) CC and b) N doped carbon.

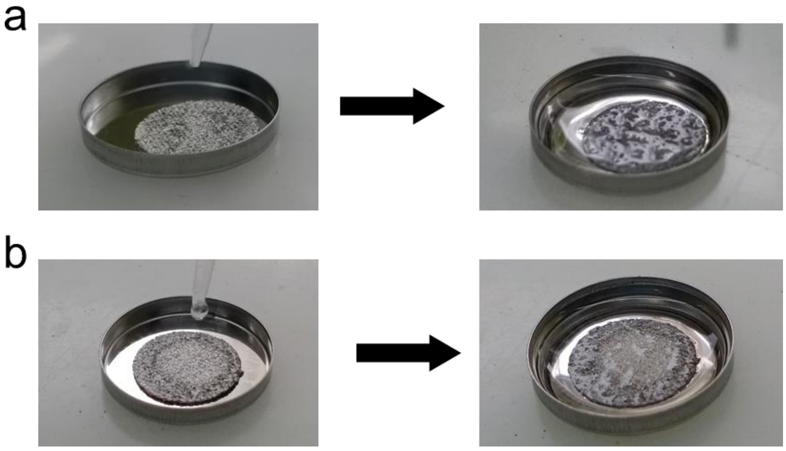

Figure S11. The leakage measurement of NaK@Co-SACN in a) PC electrolyte and b) EC:DEC electrolyte.

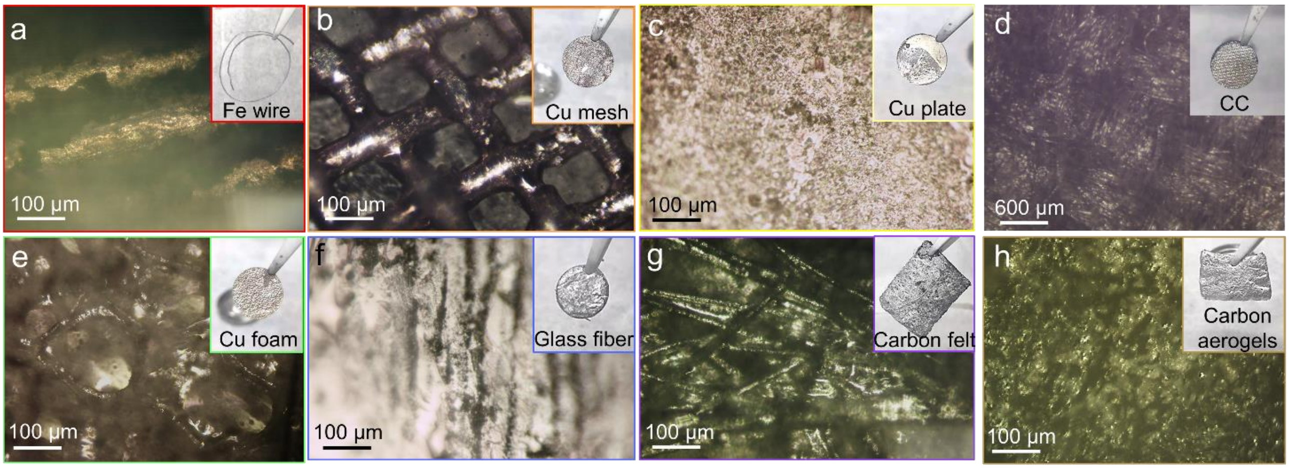

Figure S12. Optical image of Co-SACN@NaK on different substrate (a) Fe wire, (b) Cu mesh, (c) Cu plate, (d) CC, (e) Cu foam, (f) Glass fiber, (g) carbon felt, (h) carbon aerogels.

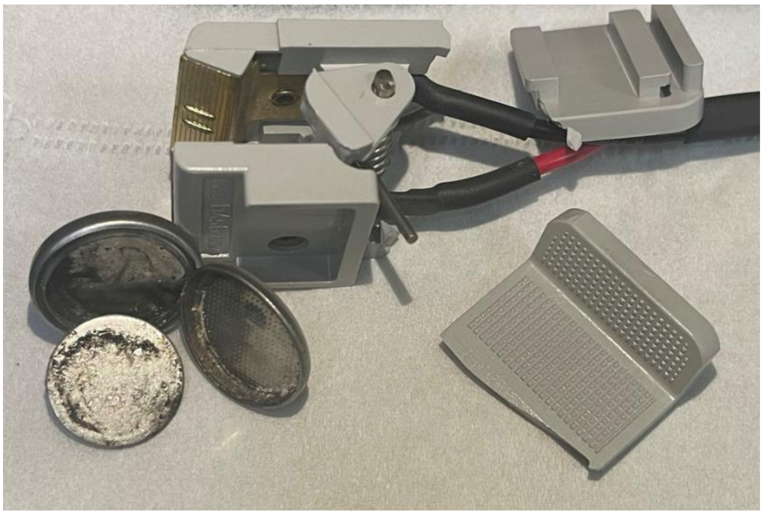

Figure S13. Optical photos Na foil battery after ~100 cycles (explode).

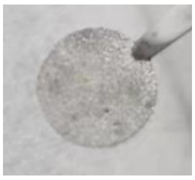

Figure S14. Optical photos NaK alloy after cycling.

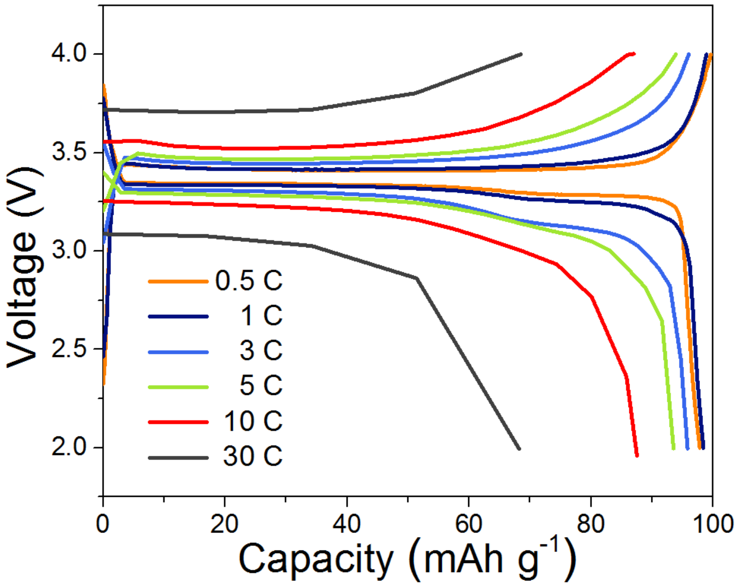

Figure S15. Voltage profiles of Na||$Na_3V_2(PO_4)_3$ at different rate.

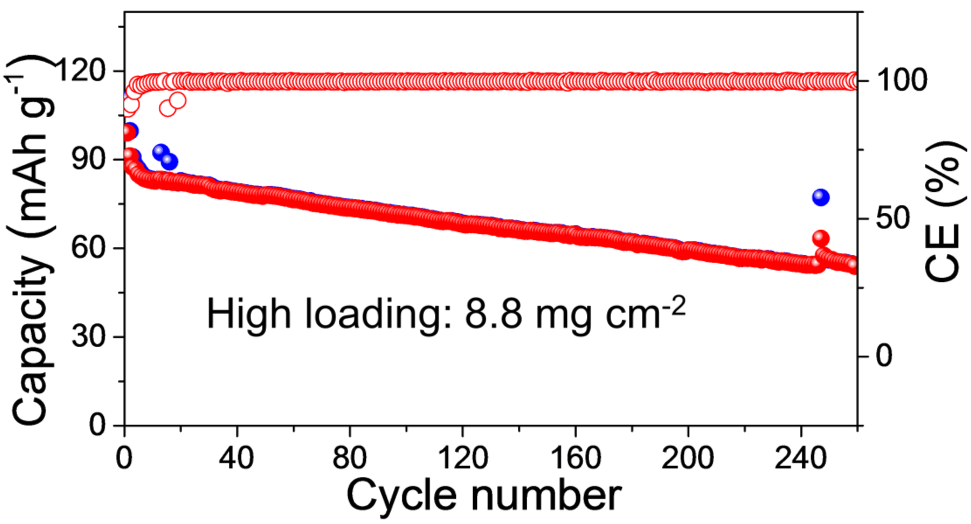

Figure S16. Cycling performance of Co-SACN@NaK‖$Na_3V_2(PO_4)_3$ with an active material of 8.8 mg cm^{-2}.

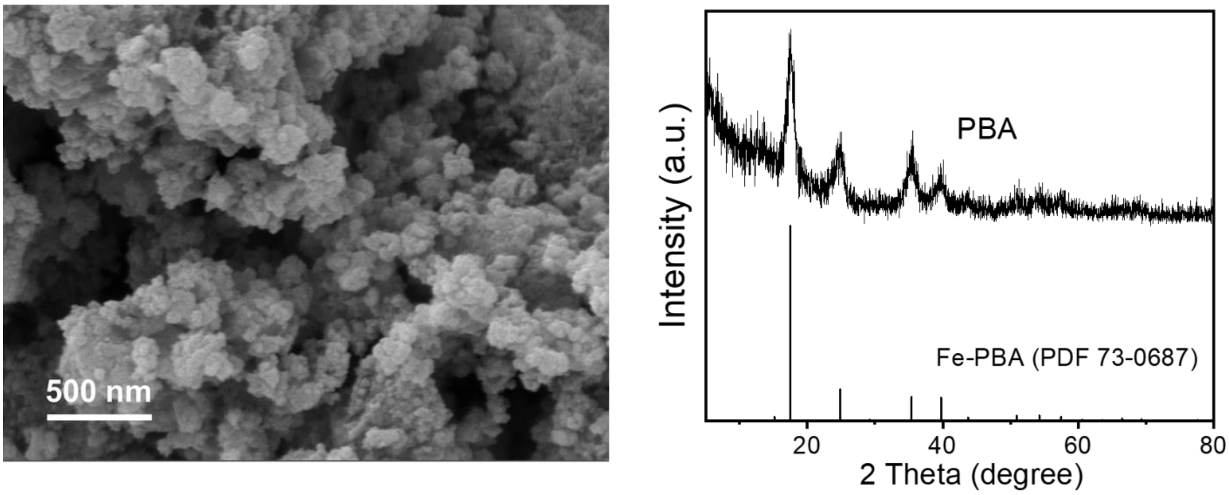

Figure S17. The characterization of PBA a) SEM, b) XRD

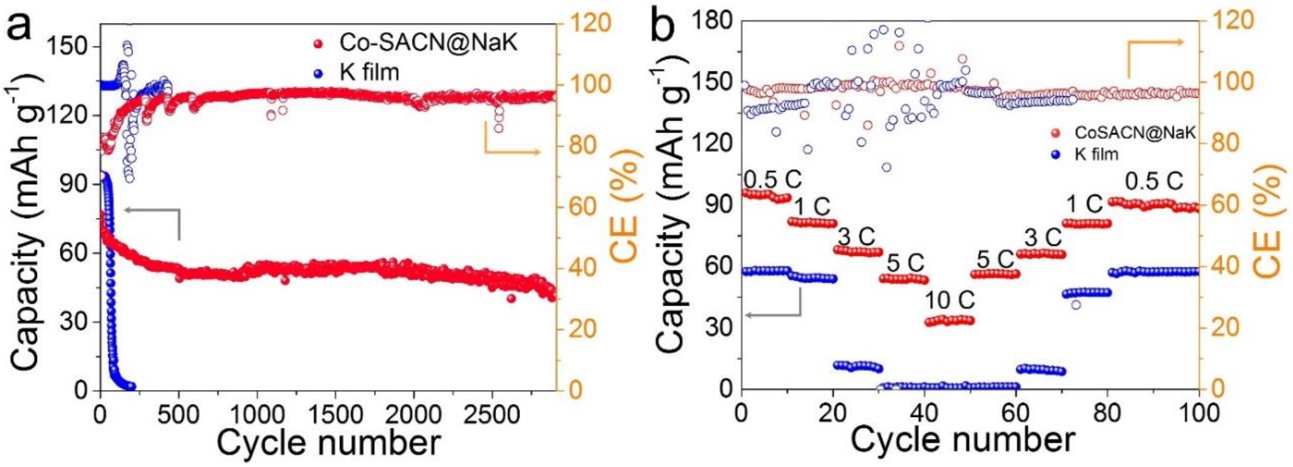

Figure S18. a) Long-term cycling performance of Co-SACN@NaK‖PBA and K‖PBA at 1 C. b) rate-performance of Co-SACN@NaK‖PBA and K‖PBA at different rate (0.5 C-10 C).

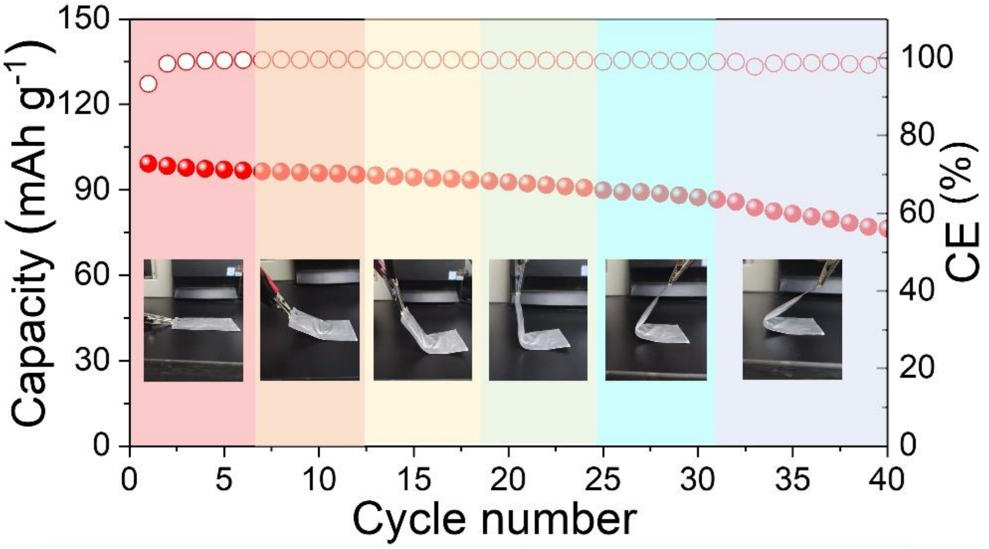

Figure S19. Cycling performance of Co-SACN@NaK‖$Na_3V_2(PO_4)_3$ pouch cell at different bending degrees.

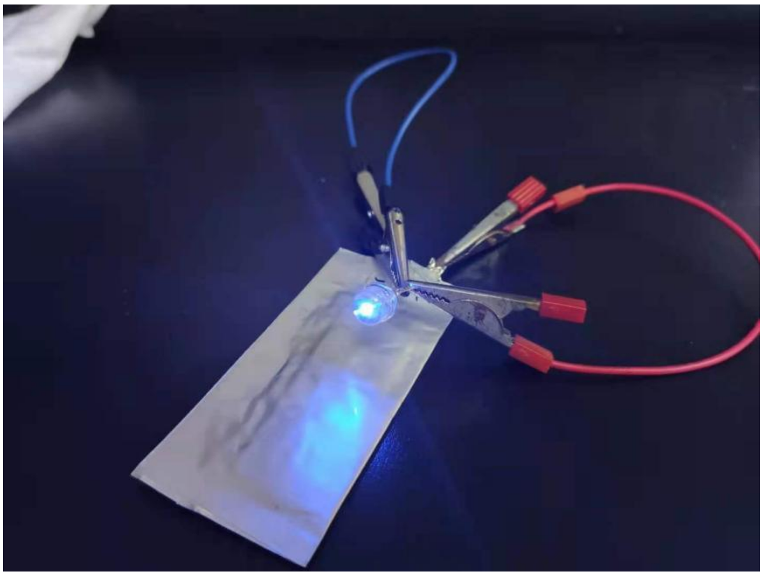

Figure S20. Digital image of pouch battery drives blue LED.

Table S1. Atomic concentration of each element in Co-SACN

Elements	Atomic concentration
C 1s	89.33
O 1s	5.78
N 1s	4.55
Co 2p	0.34

Table S1. The recent reports of Na, K and NaK based symmetric cells

Electrodes	Current density	Over-Potential	Lifespan	Ref.
Na@mld300C	(0.1 mA cm^{-2}, 0.1 mAh cm^{-2})	~350 mV	475 h	1
NaK@C	(0.4 mA cm^{-2}, 0.2 mAh cm^{-2})	~300 mV	240 h	2
CFC/KOL@Na–K	(0.4 mA cm^{-2}, 0.2 mAh cm^{-2})	~280 mV	143 h	3
K–Na–C	(0.4 mA cm^{-2}, 0.8 mAh cm^{-2})	~180 mV	2800 h	4
SAF-Na	(0.5 mA cm^{-2}, 0.25 mAh cm^{-2})	~30 mV	650 h	5
Na-wood	(1 mA cm^{-2}, 1 mAh cm^{-2})	~100 mV	500 h	6
Na/NSCNT	(1 mA cm^{-2}, 1 mAh cm^{-2})	~50 mV	500 h	7
Na-K-Sn@mesh	(2 mA cm^{-2}, 1 mAh cm^{-2})	~200 mV	550 h	8
CC@NaK	(2 mA cm^{-2}, 2 mAh cm^{-2})	~200 mV	1800 h	9
KNA-3.5	(4 mA cm^{-2}, 1 mAh cm^{-2})	~90 mV	2000 h	10
Co-SACN@NaK	(10 mA cm^{-2}, 10 mAh cm^{-2})	~250 mV	1010 h	***This work***
rGO@3D-Cu	(0.5 mA cm^{-2}, 0.5 mAh cm^{-2})	~360 mV	200 h	11
K@DN-MXene/CNT	(0.5 mA cm^{-2}, 0.5 mAh cm^{-2})	~150 mV	300 h	12
PM/NiO/K	(0.4 mA cm^{-2}, 0.2 mAh cm^{-2})	~100 mV	200 h	13
PPS treated K	(4 mA cm^{-2}, 4 mAh cm^{-2})	~250 mV	420 h	14
AlF_3@PP	(5 mA cm^{-2}, 1 mAh cm^{-2})	~200 mV	240 h	15
Co-SACN@NaK	(10 mA cm^{-2}, 10 mAh cm^{-2})	~230 mV	4000 h	***This work***

References

1 S. Zhang, Y. Zhao, F. Zhao, L. Zhang, C. Wang, X. Li, J. Liang, W. Li, Q. Sun, C. Yu, J. Luo, K. Doyle-Davis, R. Li, T.-K. Sham, X. Sun, *Adv. Funct. Mater.*, **2020**, 30, 2001118.

2 J. Yang, X. Wang, S. Huang, X. Zhang, J. Chen, *ACS Appl. Mater. Interfaces* **2021**, *13*, 40118-40126.

3 L. Zhang, X. Xia, Y. Zhong, D. Xie, S. Liu, X. Wang, J. Tu, *Adv. Mater.* **2018**, *30,* 1804011.

4 L. Xue, H. Gao, W. Zhou, S. Xin, K. Park, Y. Li, J. B. Goodenough, *Adv. Mater.* **2016**, *28,* 9608.

5 Z. Xu, J. Yang, T. Zhang, L. Sun, Y. Nuli, J. Wang, S.-i. Hirano, *Adv. Funct. Mater.* **2019**, *29*, 1901924.

6 W. Luo, Y. Zhang, S. Xu, J. Dai, E. Hitz, Y. Li, C. Yang, C. Chen, B. Liu, L. Hu, *Nano Lett.* **2017**, *17*, 3792.

7 B. Sun, P. Li, J. Zhang, D. Wang, P. Munroe, C. Wang, P. H. L. Notten, G. Wang, *Adv. Mater.* **2018**, *30*, 1801334.

8 J. Yang, X. Wang, S. Huang, X. Zhang, J. Chen, *ACS Appl Mater Interfaces* **2020**, *12,* 20423.

9 Y. Xie, J. Hu, Z. Zhang, *J. Electroanal. Chem.* **2020**, *856*, 113676.

10 Z. Tai, Y. Li, Y. Liu, L. Zhao, Y. Ding, Z. Lu, Z. Peng, L. Meng, G. Yu, L. Liu, *Adv. Sci.* **2021**, *8*, 2101866.

11 P. Liu, Y. Wang, Q. Gu, J. Nanda, J. Watt, D. Mitlin, *Adv. Mater.* **2020**, *32*, 1906735.

12 X. Tang, D. Zhou, P. Li, X. Guo, B. Sun, H. Liu, K. Yan, Y. Gogotsi, G. Wang, *Adv. Mater.* **2020**, *32*, 1906739.

13 Y. Li, L. Zhang, S. Liu, X. Wang, D. Xie, X. Xia, C. Gu, J. Tu, *Nano Energy* **2019**, *62*, 367.

14 J. Park, Y. Jeong, M. H. Alfaruqi, Y. Liu, X. Xu, S. Xiong, M.-G. Jung, H.-G. Jung, J. Kim, J.-Y.

Hwang, Y.-K. Sun, *ACS Energy Lett.* **2022**, *7*, 401.

15 P. Liu, H. Hao, H. Celio, J. Cui, M. Ren, Y. Wang, H. Dong, A. R. Chowdhury, T. Hutter, F. A. Perras, J. Nanda, J. Watt, D. Mitlin, *Adv. Mater.* **2022**, *34*, 2105855.
